# Sterol composition in plants is specific to pollen, leaf, pollination and pollinator

**DOI:** 10.1016/j.phytochem.2023.113800

**Published:** 2023-10

**Authors:** Samuel Furse, Carlos Martel, Abdikarim Yusuf, Gemma C. Shearman, Hauke Koch, Philip C. Stevenson

**Affiliations:** a Royal Botanic Gardens Kew, Kew Road, Richmond, Surrey, TW9 3AB, UK; b Faculty of Health, Science, Social Care and Education, Kingston University, Penrhyn Road, Kingston Upon Thames, Surrey, KT1 2EE, UK; c Natural Resources Institute, University of Greenwich, Chatham, Kent, ME4 4TB, UK

**Keywords:** Sterolomics, Phytosterol biosynthesis, Nutrient provision, Pollinators, Taxonomy

## Abstract

Sterols have several roles *in planta*, including as membrane components. Sterols are also essential nutrients for insects. Based on this, and the different functions of leaves and pollen, we tested the hypotheses that (a) the sterolome is different in leaves and pollen from the same plant, (b) pollens from wind- and insect pollinated plants comprise different sterols, and (c) sterol provision in pollen-rewarding angiosperms differs from nectar-rewarding species. A novel approach to sterolomics was developed, using LCMS to determine the sterol profile of leaf and pollen from a taxonomically diverse range of 36 plant species. Twenty-one sterols were identified unambiguously, with several more identified in trace amounts. C_29_ sterols dominated the sterolome in most plants. The sterol composition was significantly different in leaf and pollen and their main sterols evolved in different ways. The sterolome of pollen from animal- and wind-pollinated was also significantly different, but not between nectar- and pollen-rewarding species. Our results suggest that the sterol composition in different plant tissues is linked to their biological functions. Sterol composition in pollen might be driven by physical role rather than the nutrient needs of pollinating insects.

## Highlights

• The sterolome of tissues from a taxonomically diverse range of plants were profiled.
• Sterol metabolism in plants is generally focused on C_29_ and C_30_ sterols.
• The sterolome of leaves and pollen differ within plants.
• The sterolome of pollens from wind- and animal-pollinated species differ.
• No evidence that sterol metabolism has evolved to support insect pollinators.

## Graphical abstract

**Figure:**
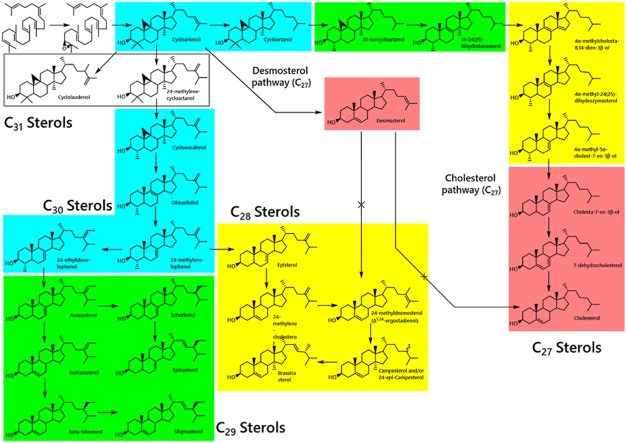

## 1 Introduction

**Fig. 1:**
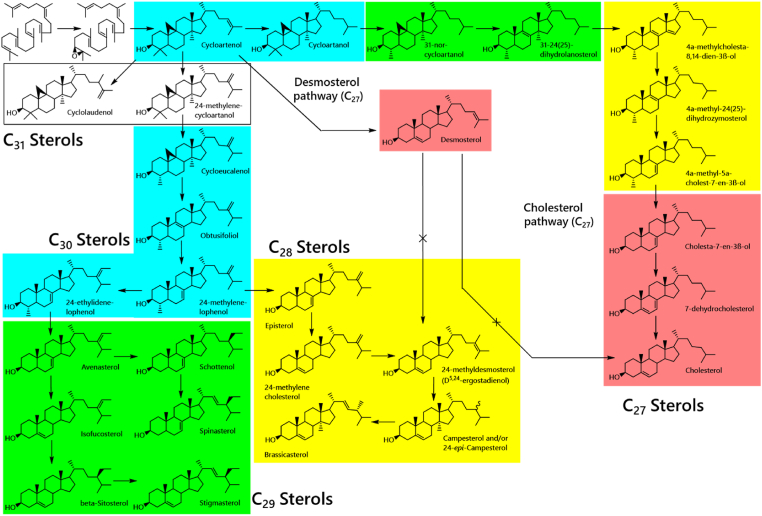
Simplified scheme of sterol biosynthetic pathways in plants, drawn from several sources (Fujimoto et al., 2000; Ghisalberti et al., 1969; Misso and Goad, 1983; Morikawa et al., 2006; Sonawane et al., 2016; Sucrow and Radüchel, 1971; Villette et al., 2015). This body of literature also shows that sterols that appear to be intermediates in the overall scheme are accumulated in some species but not others. This suggests that sterols have a range of functions in plants but also that control of sterol biosynthesis is careful, *i.e.* the result of several selection pressures.

Plants produce a remarkable variety of sterols. Sterol biosynthesis in plants comprises at least four pathways and several branches, indicating the presence of a considerable number of phytosterols present in plants (Fig. 1, constructed from several literature sources (Fujimoto et al., 2000; Ghisalberti et al., 1969; Misso and Goad, 1983; Morikawa et al., 2006; Sonawane et al., 2016; Sucrow and Radüchel, 1971; Villette et al., 2015)). These pathways show that addition and removal of single carbon atoms and desaturation occur at a few different points, explaining much of this range of sterols. The wide range of sterols made by plants is not matched by mammals, nor even by insects that feed exclusively on plant tissues (Clayton, 1964; Ling and Jones, 1995). These observations suggest that sterols have several carefully tuned but probably unconnected roles *in planta*, raising questions about how sterol biosynthesis is controlled in plants and what has influenced its evolution.

One known role of sterols in plants and other eukaryotes is as membrane components. Biophysical studies of phytosterols in model membrane systems using calorimetry and resonance energy transfer have shown that for membranes with low relative proportions of sterol, *beta-*sitosterol (C_29_), campesterol (C_28_) and brassicasterol (C_28_) all lower the gel/fluid transition temperature of the model membrane more than cholesterol (C_27_) (Benesch and McElhaney, 2014; Halling and Slotte, 2004). A fluorescence study has shown that campesterol is the most ordering of cholesterol, campesterol and *beta-*sitosterol (Grosjean et al., 2015). However, the concentration of sterol in the membrane also affects the nature of the gel/fluid transition, with sterol-rich domains in the membrane becoming increasingly more prevalent than sterol-poor domains in the membrane at higher sterol concentrations. Additionally, while the temperature of the gel/fluid phase transition decreased for the sterol-poor domains with increasing overall sterol abundance, the reverse was true for the sterol-rich domains (Benesch and McElhaney, 2014; Halling and Slotte, 2004). This suggests that sterol profile, concentration and lateral distribution in the membrane are important for understanding how the membrane will respond to changes in temperature and that several spatial, synthetic and compositional factors surrounding phytosterols could be exploited to control the response of membranes to temperature.

In addition to the physical roles of sterols in plants, plants are also a source of sterols for herbivores, including pollinating insects. Sterol provision has long been thought part of a mutualistic relationship; plants rely on pollinators for reproduction and pollinators, particularly bees, rely on plants for the dietary supply of sterols. In fact, all insects are auxotrophic for sterols. Studies of honeybee nutrition have shown that honeybees require 24-methylenecholesterol (24MC) as their bulk sterol and need small amounts of campesterol and cholesterol to produce moulting hormones (Review, Furse et al., 2023). The nutritional need of pollinators for sterols from plants suggests that a selection pressure may have or does exist for plants to adapt their sterol provision to support pollinators. However, this is separate to the role of sterols as membrane components and also as intermediates in the biosynthesis of defence compounds such as saponins, suggesting a compromise between selection pressures may also exist.

The biochemical and biophysical evidence collected to date shows that sterols are essential to both plants and insects, and may form part of the relationship between them. The importance of good nutrient provision to pollinators becomes clear when we consider that approximately 90% of angiosperms depend upon animals for reproduction (Rodger et al., 2021) including 75 of the 100 of the most important arable crops (Ollerton et al., 2011). However, the sterol composition of pollen from angiosperms differs considerably between taxonomic groups and climatic conditions, but not by the pollinator type in animal-pollinated plants (Zu et al., 2021), suggesting that adaptation to insect nutritional needs is not universal. It is not clear whether the sterol composition of pollens differs between wind- and animal-pollinated plants. Pollen in wind pollinated plants is dry compared to the sticky pollen of animal pollinated plants (Lin et al., 2013; Pacini and Hesse, 2005). Sticky pollen is associated with the presence of the pollenkitt which is mainly composed of lipids (Dobson, 1988), and is found in almost all zoophilous angiosperms (Pacini and Hesse, 2005). However, greasy compounds such as lipids and sterols are more lubricating than adhesive, so what the relationship is between the molecular composition of the pollenkitt, and pollinator type, is unclear.

Taken together, the evidence about the variety of sterol profiles in plants includes but is not limited to their function as membrane components, the range of climatic conditions they live in and nutrient provision to pollinators. In our view, several studies would be required to test all these possibilities. For the present study, the importance of a mutualistic relationship was taken as the focus. Current evidence shows that a range of sterols are found in pollen, including several that have a two-carbon group on carbon 24, which are not abundant in bees. Furthermore, there are no reports of bees comprising C_30_ sterols (*e.g.* cycloartenol), despite evidence of them in angiosperm pollen (Zu et al., 2021). This would suggest that insects evolved to the nutrient availability from plants, and that the sterol composition of plant tissues is tied more closely to their functions in the plant rather than as a source of nutrients. However, plants have evolved to provide nectar as a reward to pollinators thus plants are susceptible to pollinator-driven selection pressures. This led us to the over-arching hypothesis that plants have been influenced by the nutritional needs of pollinators.

We addressed this general hypothesis by testing three specific hypotheses. First, that pollen and leaves from the same plant have different sterol compositions. Testing this hypothesis showed whether the sterolome was tissue-specific and thus whether sterol composition was specific in pollen. Second, that pollens from wind- and animal-pollinated plants have a different sterol composition. If true, this would indicate that the selection pressures that shape the sterol composition of pollen have been driven by factors associated with pollination type. This test is important as it can tell us whether selection pressures associated with insects are possible. Third, the sterols in pollen from plants that offer pollen as a pollinator reward are different to those that offer only nectar. If the latter hypothesis is true, it would imply that the relationship between plant and pollinator has been shaped at least partly through sterol provision.

To test these hypotheses, we collected leaf and pollen samples from a taxonomically diverse range of angiosperms and two gymnosperms, extracted the sterols from them and profiled them using liquid chromatography-mass spectrometry (LCMS). We then used a combination of statistical tests and ancestral reconstruction to identify potential differences in sterol profile, which sterols drove any difference between groups and how they have evolved.

## 2 Results

### 2.1 Sterolome of plants

**Fig. 2:**
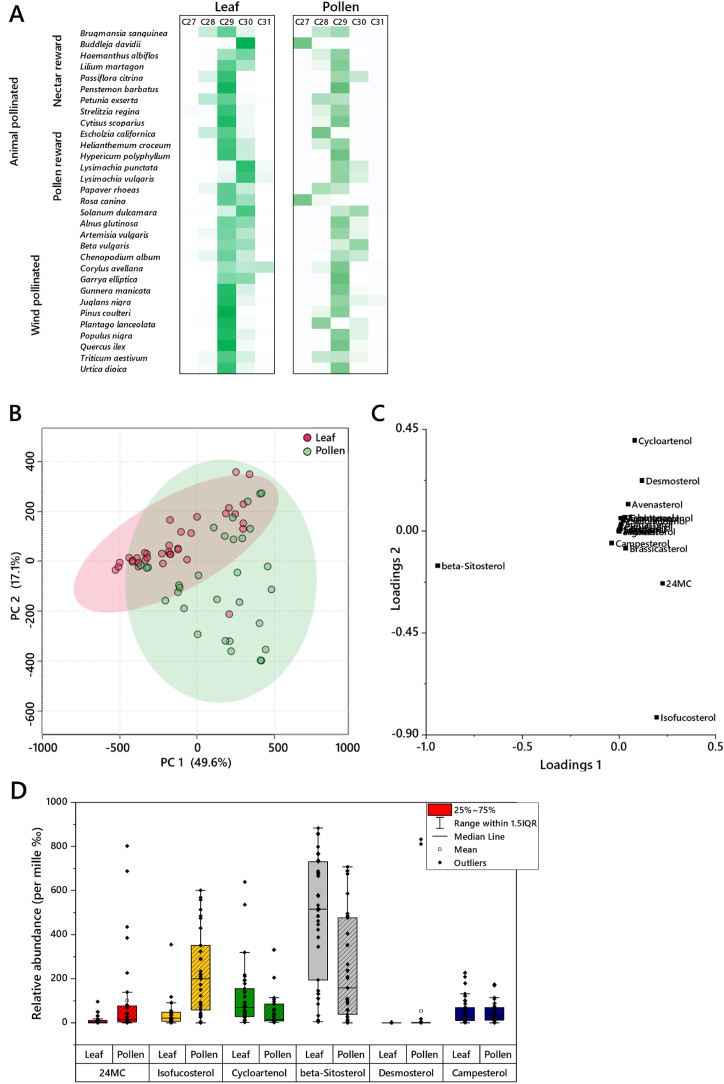
The distribution of leaf and pollen samples according to their sterol composition. Panel **A**, heatmap diagram of the relative abundance of C_27_, C_28_, C_29_, C_30_ and C_31_ sterols in leaf and pollen samples, by pollination type. Panel **B**, Principal Component Analysis (PCA) plot. Coloured areas show 95% coverage. Panel **C**, loadings plot for the PCA. Panel **D**, The abundance of the phytosterols that distinguish pollen and leaf tissues, shown as box-and-whisker plots. These variables account for the amount of the difference with the following proportions: beta-Sitosterol (25%), isofucosterol (14%), cycloartenol (8%), 24-methylenecholesterol (7%), campesterol (3%).

A basic pathway analysis in which the sterols were grouped by the number of carbons (sterol metabolism shown in Fig. 1, groupings in Table 2) showed that although the sterol composition varies considerably in plants, generally C_29_ sterols dominate the sterol profile (>60% of total sterol signal, Table S1) in both leaves and pollen in the species surveyed. (The C_29_ group comprises *beta*-sitosterol, isofucosterol and avenasterol; Fig. 1, Table 2). In the plants for which paired pollen and leaf samples were collected, C_29_ sterols dominated in the pollen of wind-pollinated plants in 12 of the 14 species tested, whereas C_29_ sterols dominated in 10 of the 16 animal-pollinated species tested (Fig. S1). The pollen from animal pollinated species could also be dominated by C_27_ sterols (two species sampled) which were very low in the sterolome of pollen from wind pollinated plants (Fig. S1). Some plants typically have an approximately-equal mixture of C_28_ and C_29_ sterols or C_29_ and C_30_ sterols (*e.g.* *Alnus glutinosa* leaves, *Petunia exserta* pollen, Fig. 2A).

### 2.2 Sterol composition within plants

To test the hypothesis that the plant controls sterol provision specifically in pollen, we determined whether leaf and pollen have different sterolomes. If true, this would show that sterol composition was both controlled and tissue-specific. Principal Component Analysis (PCA, Fig. 2B) showed some differences, with several sterols driving the differences observed (Fig. 2C). PERMANOVA results after controlling for species identity showed that pollen and leaves have different sterol compositions (pseudo-F_1,78_ = 9.46, *p* < 0.0001). SIMPER tests indicate that five sterols, *beta*-sitosterol, isofucosterol, 24MC, desmosterol, cycloartenol, drove 60% of the difference between them. The C_29_ sterols beta-sitosterol (25%), isofucosterol (14%) were the highest-ranked sterols. Statistical plots of these sterols (Fig. 2D) show that the abundance of these sterols vary in different ways; isofucosterol is more abundant in pollen whereas *beta*-sitosterol is more abundant in leaves. This shows that either the way sterols are transported around the plant differs between tissues or that the de novo biosynthesis differs between tissues.

Comparison of the leaf and pollen profiles using a heatmap (Fig. 2A) suggests that the sterol profile of pollen may also be more varied across taxa than that of leaves. This variety across taxa was unexpected because both tissues have roles fundamental to the survival of the plant (photosynthesis and reproduction). This led us to ask how the sterol composition in pollen and leaves emerged. To do this, we reconstructed the ancestral phylogeny of the most important sterols in species profiled.

**Fig. 3:**
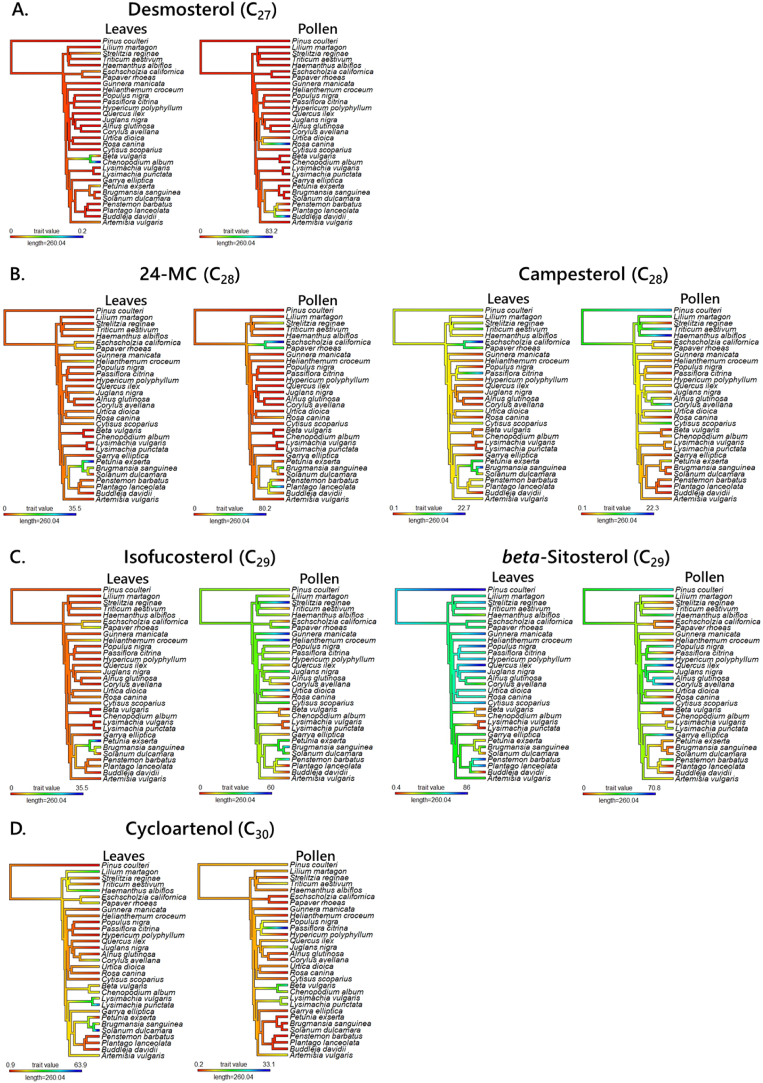
Ancestral reconstruction based on sterol composition of the most abundant sterols in leaf and pollen samples. Panels **A-D**, represent C_27_, C_28_, C_29_ and C_30_ sterols respectively. The colour range denotes the percentage of that sterol (relative abundance) in each tissue within the relative abundance range of each sterol type. The ancestral hierarchy indicates the relationship between sampled species. (For interpretation of the references to colour in this figure legend, the reader is referred to the Web version of this article.)

The ancestral phylogeny of the six sterols that describe the biggest differences between the two tissues is shown in Fig. 3. This analysis showed that, like the analyses above, closely related species are more similar in sterol composition than less closely related ones. Furthermore, the ancestral state of the relative sterol composition was similar for most of the basal angiosperm clades with only relatively narrow changes in individual groups. Differentiation of the sterol abundance at the tips appears to be quick and strong in most of the studied taxa. Taken together, these results suggest that the seterol-based selection pressures experienced by leaves and pollen were different. These results support the hypothesis that local biosynthesis or accumulation of sterols differs between tissues and that this is driven by evolutionary changes below family level.

This analysis suggests that tissues in which 24-methylenecholesterol (24MC) dominates were unconnected as well as relatively rare. This is surprising as generalist bee pollinators use 24MC as their bulk sterol and are dependent on pollen for 100% of their dietary supply. 24MC appears at or below family level only, with only a handful of species having >5% in their sterol fractions. This does not support the hypothesis that nutritional needs of pollinators that consume pollen have provided a selection pressure that drove accumulation of 24MC in pollen. In order to investigate whether there were general pressures, we tested the hypothesis that sterol composition of pollen differed between wind- and animal-pollinated plants, and then tested whether sterol provision differed between nectar-rewarding and pollen-rewarding angiosperms.

### 2.3 Sterol composition of pollen from wind- and animal-pollinated plants

**Fig. 4:**
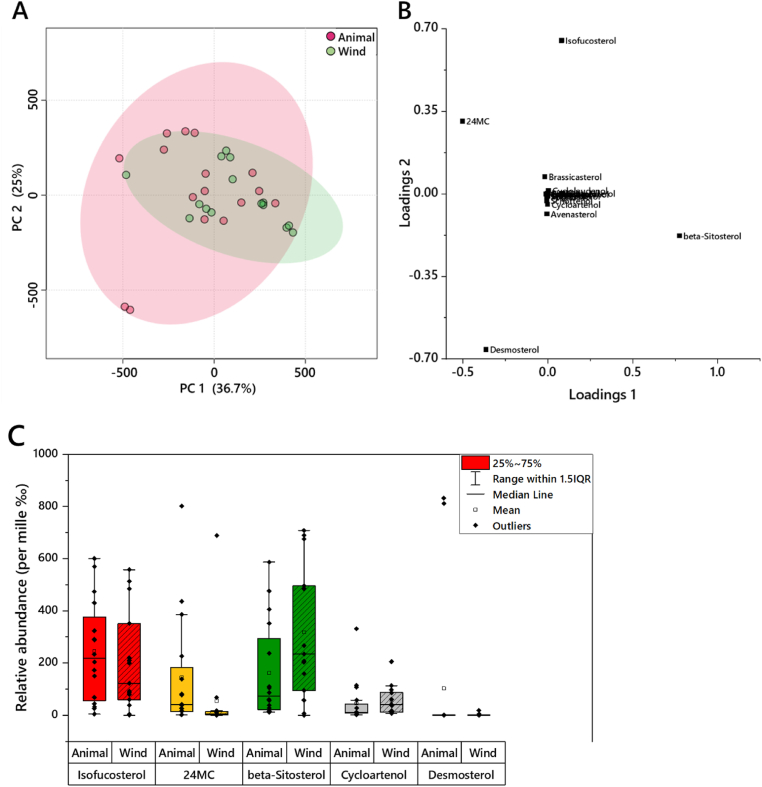
The distribution of pollen samples from animal- and wind-pollinated species, according to their sterol composition. Panel **A**, Principal Component Analysis (PCA) plot. Coloured areas show 95% coverage. Panel **B**, Loadings plot for the PCA. Panel **C**, the phytosterols that distinguish the pollen from animal- and wind-pollinated species, shown as box-and whisker plots. The y axis is relative abundance (per mille). These variables account for the amount of the difference with the following proportions: *beta*-Sitosterol (19%), isofucosterol (15%), 24-methylenecholesterol (11%), desmosterol (7%), cycloartenol (5%).

We used a PERMANOVA to test the hypothesis that pollen from wind- and animal-pollinated plants differ. This test showed that here too, the sterol composition of the wind- and animal-pollinated plants differed; pseudo-F_1,34_ = 2.17, *p* < 0.05 (see PCA in Fig. 4). SIMPER indicated this effect was driven by four major sterols, with C_29_ sterols beta-sitosterol and isofucosterol accounting for over 30% of the difference between the groups (Fig. 4). We also tested whether total sterol in pollen (mg/Kg pollen) was different in wind- and animal-pollinated species, but found no effect (PERMANOVA: pseudo-F_1,34_ = 1.28, p = 0.27). We also found no effect of taxonomic order on sterol composition (PERMANOVA: pseudo-F_18,34_ = 1.25, *p* = 0.13).

Sterol composition of pollen from plants that offer pollen as a reward and those that offer only nectar.

The hypothesis that the sterol composition of pollen from nectar-rewarding plants is different to pollen-rewarding species was tested by comparing the sterol compositions of pollen from several plants representing each. This is a useful test as the expectation is that sterol-based selection pressures to affect the pollen-reward plants but not the nectar-reward plants. Comparison of the sterol composition of plants with different floral rewards showed that there was a difference in sterol composition between them (PERMANOVA: pseudo-F_2,17_ = 5.01, *p* = 0.01), specifically when comparing oil-rewarding plants against nectar- and pollen-rewarding plants (post hoc test: *p* < 0.05). Similarly, we found that the sterol composition has been affected by the pollinator type (PERMANOVA: pseudo-F_4,17_ = 4.98, *p* = 0.01). The sterol profile of bee-pollinated plants was different to generalist- and hummingbird-pollinated plants (post hoc tests: *p* < 0.05), whereas it was barely significant when comparing to butterfly and moth pollinated taxa (*post hoc* tests: 0.1> *p* > 0.05). We also found a strong effect of the taxonomic relatedness in the sterol composition among the sampled animal-pollinated plants (PERMANOVA: pseudo-F_9,17_ = 4.69, *p* = 0.01).

## 3 Discussion

In this study we investigated how the sterolome of pollen and leaves has evolved. Testing our hypotheses revealed that sterol composition is tissue specific and pollination-type-specific, but may not be nutrient-reward specific. An ancestral phylogenetic reconstruction showed that selection pressures that affected sterol composition in pollen generally existed at or below family level, consistent with evidence for fundamental roles of sterols in plants that are not specific to pollination.

The evidence for tissue-specific sterol composition shows that *de novo* biosynthesis of sterols and/or accumulation is indeed controlled in the plant and probably local. This in itself is not surprising as tissues such as leaves and pollen have markedly different functions and thus one would expect the selection pressures they have experienced to be different. However, exactly what the roles of sterols are and how the needs of distinct tissues differ is unclear. Plants face a complicated set of climatic conditions with little to defend them; the temperature of membranes in warm-blooded organisms such as mammals is carefully controlled. Cholesterol is an important membrane component in such vertebrates, influencing the physical properties of those membranes (review McMullen et al., 2004)), suggesting that one sterol is sufficient for retaining membrane activity in these organisms. However, plants face a much greater range of temperatures; the mid-temperate UK saw ground temperatures of −8 °C and 40 °C in 2022. Changes of temperature of >10 °C within 24 h are also typical throughout the year. The range of sterols present in plants, and the greater emphasis on more fluidising sterols, may therefore be an adaptation to the need for plant membranes to remain fluid despite low and changing temperatures.

The possibility that sterol metabolism has evolved primarily to service maintenance of membrane properties suggests that in evergreen plants in particular, membranes must be flexible and resistant to changes in temperature. This is also observed anecdotally through regions; there does not appear to be an important difference between tropical and temperate species in the ancestral reconstruction in this study, suggesting that global region has not influenced sterol composition. Sterol biosynthesis and the lateral distribution of sterols in membranes in plants may therefore be organised to serve complex structural needs.

Specifically, the biophysical work done to date has shown that sterols with an alkyl group on C24 of the sterol skeleton such as campesterol and *beta*-sitosterol (Fig. 1) have a more fluidising effect on membranes that cholesterol, which has no alkyl group on C24. However the concentration of sterol in membranes has also been found to be important (Benesch and McElhaney, 2014; Grosjean et al., 2015; Halling and Slotte, 2004), suggesting that several sterol parameters can be used to modulate membrane behaviour. This might explain the different sterol compositions of pollen and leaves; leaves are required to exist for longer periods than pollen and to resist a wider range of temperatures. The pollen is also required to grow a pollen tube, requiring a different geometry of membrane to typical pseudo-spherical cells. These observations provide an explanation for the different sterol compositions of the two different tissues, however selection pressures imposed by other factors are also important. The relationship between plants and insect pollinators is equally important to species survival in angiosperms as animal pollinators are key for plant reproduction and thus survival. One hypothetical selection pressure is the nutrient needs of pollinators.

Insects are auxotrophic for all sterols, meaning they are unable to make the sterol skeleton or modify the structure of sterols they consume. The supply of sterols insect pollinators need must always have been sufficient, at least for the lineages that remain. This implies that the plants have evolved to satisfy the insects' needs or that insects have evolved to what is available, or both. These possible selection pressures would be expected to interact with the structural roles of sterols that serve the plants’ needs for daily survival (changes in temperature) and the physical nature of specialist processes (pollen tube formation). Importantly, both C_28_ and C_29_ sterols (Fig. 1) are made from the same intermediate, 24-methylenelophenol, with a one-step methylation required to produce C_29_ sterols. Thus, if a selection pressure were applied to favour C_28_ sterols, a loss, blocking or other mutation of only one gene would be required to favour the C_28_ pathways. As this has not happened, we must assume that biosynthesis of C_29_ sterols is more important to plants than C_28_.

Data from the current study shows that the majority of plants were dominated by C_29_ or C_30_ sterols (Fig. 2A), and that although there is a clear difference between both leaf and pollen, and wind- and animal pollinated plants, this is not at all consistent with sterols found in pollinators, especially generalist bees. This strongly suggests the plants’ physical needs outweigh the selection pressures exerted by the nutritional needs of insects. This is consistent with insects having evolved to nutrient availability rather than plants having evolved to pollinator need.

However, where pollen is provisioned as a reward it must be nutritionally valuable to pollinators. This informed our hypothesis that nectar- and pollen-rewarding plants would offer different sterols. The tests of this hypothesis in the current study did not show convincingly that there was a difference in sterol composition between these two groups. However, the groups were relatively small (*n* = 8) and plants such as *Buddlia davidii* and *Lysimachia* spp. are generalist plants that provide pollen and nectar or oil to pollinators. This suggests that a larger sample size (greater statistical power) and more careful subdivision by reward type may be required in order to reveal any difference in sterol provision by these plants. However, as the phylogenetic reconstruction shows that the few plants whose pollen sterolome is dominated by 24MC are typically unconnected, our suggestion is that any pattern that was observed would be a convergent rather than taxonomically-driven effect.

The presence of sterols in pollen that bees do not contain or need suggests one other possibility, that plants use sterols as defence compounds. Sterols are used to produce saponins, and they are well established as specific defence compounds. It is not clear from our data whether sterols are used for defence by plants, however further study using foraging data, *i.e.* whether bees favour pollen with the sterols they need in it and avoid others, might reveal this.

## 4 Conclusions

This study has shown that the sterol biosynthesis is under careful control in species across the plant kingdom, with evidence for differences between pollination modes. However, the hypothesis that pollen from nectar-rewarding and pollen-rewarding species comprised different sterols was not supported. This suggests that the selection pressures applied to plants that have shaped the sterol composition of their pollen have not generally been driven by the nutrient demand of insects.

The role of the sterols in pollen is therefore expected to be more physical, probably as a membrane component. This may include the membrane expansion done during the formation of the pollen tube. Specifically, we suggest the need for C_29_ and C_30_ sterols in most plants has been the result of a selection pressures based on structural needs of both leaf and pollen tissues and not nutrient demand by pollinating insects such as bees.

## 5 Experimental

**General experimental procedures.** Solvents and fine chemicals were purchased from Sigma-Aldrich (Gillingham, Dorset, UK) and not purified further, with the *d*_*7*_-Cholesterol used as internal standard obtained from Avanti polar lipids (Alabama, USA). Plasticware was bought from Sarstedt (Darmstadt, Germany) or Fisher Scientific (Herfordshire, UK).

**Plant species.** Plant samples were collected from RBG Kew or domestic gardens in West London (May–August 2022). Forty species were sampled, from 24 families and 21 orders. The plant species sampled are shown in Table 1.

**Table 1:** The list of plant species used in the present study. Where both pollen and leaf samples were collected, they were collected from the same individual plant. Only samples with paired pollen and leaf were used in the ASR.

Species	Family	Order	Sample type (1 = pollen and leaf, 2 = leaf only, 3 = pollen only)	Paired leaf and pollen samples (Y/N)	Wind/animal (1 = animal, 2 = wind)	Nectar/Pollen reward (NA = not applicable, 1 = nectar, 2 = pollen
*Alnus glutinosa* (L.) Gaertn.	Betulaceae	Fagales	1	Y	2	NA
*Araucaria araucana* (Molina) K.Koch	Araucariaceae	Araucales	2	N	2	NA
*Artemisia vulgaris* L.	Asteraceae	Asterales	1	Y	2	NA
*Beta vulgaris* L.	Amaranthaceae	Caryophyllales	1	Y	2	NA
*Brugmansia sanguinea* (Ruiz & Pav.) D.Don	Solanaceae	Solanales	1	Y	1	1
*Buddleja davidii* Franch.	Scrophulariaceae	Lamiales	1	Y	1	1
*Buddleja officinalis* Maxim.	Scrophulariaceae	Lamiales	2	N	1	1
*Chenopodium album* L.	Amaranthaceae	Caryophyllales	1	Y	2	NA
*Chenopodium* sp.	Amaranthaceae	Caryophyllales	2	N	2	NA
*Corylus avellana* L.	Betulaceae	Fagales	1	Y	2	NA
*Cytisus scoparius* (L.) Link	Fabaceae	Fabales	1	Y	1	2
*Dactylis glomerata* L.	Poaceae	Poales	2	N	2	NA
*Eschscholzia californica* Cham.	Papaveraceae	Ranunculales	1	Y	1	2
*Fuchsia denticulata* Ruiz & Pav.	Onagraceae	Myrtales	2	N	1	1
*Garrya elliptica* Douglas ex Lindl.	Garryaceae	Garryales	1	Y	2	NA
*Gunnera manicata* Linden ex André	Gunneraceae	Gunnerales	1	Y	2	NA
*Haemanthus albiflos* Jacq.	Amaryllidaceae	Asparagales	1	Y	1	1
*Helianthemum croceum* (Desf.) Pers.	Cistaceae	Malvales	1	Y	1	2
*Helix hedera* L.	Araliaceae	Apiales	3	N	1	1
*Hibbertia grossulariifolia* (Salisb.) Salisb.	Dilleniaceae	Dilleniales	2	N	1	2
*Hypericum polyphyllum* Boiss. & Balansa	Hyperiaceae	Malpighiales	1	Y	1	2
*Juglans nigra* L.	Juglandaceae	Fagales	1	Y	2	NA
*Lilium martagon* L.	Liliaceae	Liliales	1	Y	1	1
*Lysimachia punctata* L.	Primulaceae	Ericales	1	Y	1	2
*Lysimachia vulgaris* L.	Primulaceae	Ericales	1	Y	1	2
*Papaver rhoeas* L.	Papaveraceae	Ranunculales	1	Y	1	2
*Passiflora citrina* J.M.MacDougal	Passifloraceae	Malpighiales	1	Y	1	1
*Penstemon barbatus* (Cav.) Roth	Plantaginaceae	Lamiales	1	Y	1	1
*Petunia exserta* Stehmann	Solanaceae	Solanales	1	Y	1	1
*Pinus coulteri* D.Don	Pinaeceae	Pinales	1	Y	2	NA
*Plantago lanceolata* L.	Plantaginaceae	Lamiales	1	Y	2	NA
*Populus nigra* L.	Salicaceae	Malpighiales	1	Y	2	NA
*Quercus ilex* L.	Fagaceae	Fagales	1	Y	2	NA
*Rosa canina* L.	Rosaceae	Rosales	1	Y	1	2
*Rumex sanguineus* L.	Polygonaceae	Caryophyllales	3	N	2	NA
*Salix* sp.	Salicaceae	Malpighiales	3	N	2	NA
*Solanum dulcamara* L.	Solanaceae	Solanales	1	Y	1	2
*Strelitzia reginae* Banks	Strelitziaceae	Zingiberales	1	Y	1	1
*Triticum aestivum* L.	Poaceae	Poales	1	Y	2	NA
*Urtica dioica* L.	Urticaceae	Rosales	1	Y	2	NA

**Table 2:** The grouping of sterols by the number of carbon atoms. Only those identified sterols are listed.

	C_27_		C_28_				C_29_				C_30_			C_31_
Isoform	ST(27:1)	ST(27:2)	ST(28:0)	ST(28:1)	ST(28:2)	ST(28:3)	ST(29:0)	ST(29:1)	ST(29:2)	ST(29:3)	ST(30:1)	ST(30:2)	ST(30:3)	ST(31:2)
*m/z*	369.352	367.336	385.383	383.367	381.351 399.362	379.336	399.398	397.383	395.367 413.378	393.351	411.399	409.383 427.394	407.367	423.399 441.409
Name	Chole-sterol	Desmo-sterol		Campe-sterol	Episterol 24MC Brassicasterol	Ergosterol	Sitostanol	*beta*-Sitosterol Schottenol	Isofucosterol Avenasterol Spinasterol Stigmasterol	Anthelsterol	Cyclo-artanol	Obtusi-foliol Cycloeucalenol Cycloartenol 24MCA		Cyclolaudenol

### 5.1 Plant tissue preparation

**Pollen.** Hand-collected pollen (10–20 mg, stored at −80 °C, Table 1) was doped with an internal standard (20 μL, d_7_-cholesterol in methanol at 0·1 mg/mL) and dispersed into methanolic potassium hydroxide (300 μL, 10% *w/v*) before being heated (80 °C, water bath, 1·5 h) with a septum cap on. These were then cooled, diluted with DMT (500 μL, dichloromethane: methanol: triethylammonium chloride 3:1:0·002 *v/v/w*) and water (500 μL, ddH_2_O) before being agitated briefly (fresh cap) and spun down (500 *g*, 2 min). A portion of the organic solution was transferred to a sample running vial and the solvent removed under a stream of low-oxygen, gaseous nitrogen. The resulting film was redissolved in methanol (50 μL, LCMS quality) and closed with a septum cap before data acquisition (within 48 h of preparation).

**Leaves.** Leaves were collected (∼5–10 g wet weight, Table 1) before being dried (−80 °C, 1 mBar) and disrupted mechanically. The rough-cut, dried leaf material (300–1200 mg/specimen) was then dispersed in methanolic potassium hydroxide (10% *w/v*, 15 mL) before being heated (80 °C, 15 h). The mixtures were allowed to cool (16 h) before being diluted (methanol, total liquid volume ∼15 mL) and washed with petroleum ether (4 × 5 mL). A sample (50 μL) of the combined extracts was transferred to a sample running phial (300 μL, glass insert) and dried under a stream of low-oxygen, gaseous nitrogen. The resulting film was redissolved in methanol (50 μL, LCMS quality), closed with a septum cap before data acquisition (within 48 h).

### 5.2 Data collection and processing

**Liquid Chromatography Mass Spectrometry (LCMS).** Mass spectra were acquired on a Thermo Scientific Orbitrap Fusion mass spectrometer equipped with a Atmospheric Pressure Chemical Ionisation (APCI) probe, coupled to a Thermo Scientific Vanquish liquid chromatograph stack (Thermo Scientific, UK).

The chromatography was conducted using a Thermo Scientific Hypersil GOLD LCMS C_18_ column, 50 × 2·1 mm, particle size 1·9 μm (ThermoScientific, UK) held at 55 °C for all samples. The injection volume was 1 μL with a mobile phase flow rate of 0·60 mL/min. Eluents were A, methanol (LCMS grade); B, water (double-distilled, deionised). The method for chromatographing sterols was 16% B (0–11 min), 0% B (11·1–16·4 min), 16% B (16·5–19 min). The total running time was 20 min per sample, including sample changeover and a washing step.

Mass spectrometric data were collected in positive ionisation mode by APCI using a resolution of 120 000, a spray voltage of 2·86 kV, sheath nitrogen gas flows of 45 (sheath), 5 (auxiliary) and 1 (sweep) arbitrary units, and ion transfer tube and vaporizer temperatures of 300 °C and 350 °C. The mass acquisition window was *m/z* 197–500, the low mass being set to capture the fluoranthene cation (*m/z* 202.077) used for internal mass calibration.

Using this system and conditions, the internal standard used for samples (*d*_*7*_-cholesterol) eluted at 5.6 min as the main ion species, [M-H_2_O + H^+^)]^+^ at *m/z* 376·395, was measured within a mass variance of <1 ppm. The external references used, along with their main ions, masses and retention times are listed in Table S2.

**Preparation of the sample run.** Samples, blanks, reference samples and QCs were prepared to be run in one sequence, with a blank sample run first. The leaf and pollen samples were randomised within themselves, with blank and QC samples dispersed randomly throughout the sequence and the set of external stock reference samples run before and after the pollen and leaf samples. The autosampler temperature was 15 °C for the whole run. Solvent blanks (LCMS-quality methanol only; 7 blanks were dispersed randomly through the run) and extraction blanks (comprising *d*_*7*_-cholesterol, 3 blanks dispersed randomly through the run) were included along with QCs (commercially available polyfloral pollen, 2·5, 5·0 and 10 mg loadings, three each).

**Data processing.** Data files (*.raw, 50–60 MB ea.) were processed using Analyzer PRO XD (SpectralWorks Ltd, Cheshire, UK). Signals were extracted from 3 to 11 min in a mass range of 360–442 *m/z*, with an area threshold of 500 000, smoothing of 15 arbitrary units, a width of 0.01 min, mass accuracy of 3 d. p. and a retention time window of 0.1 min. The combination of measured *m/z* and retention time (min, 3 d. p.) was used for all signals (referred to here as a level 3 annotation). Signals with an average size 3 × higher than the average size of that signal in the blanks) with an R_T_ 3–11 min were given a level 2 annotation (format ST(27:1) where ST represents ‘sterol’, the first number in parentheses is the number of carbons and the second number is the number of double-bonds). Compounds were assigned names (a level 1 annotation) only where stock reference materials that had been verified using multi-nuclear NMR were available as reference samples: 24-methylenecholesterol, 24-methylenecycloartenol, anthelsterol (a sterol found in *C. anthelmintica*, ST(28:3), that is active under 330 nm u. v. but whose structure has not been determined formally using NMR), avenasterol, *beta*-sitosterol, brassicasterol, campesterol, cholesterol, cycloartenol, cycloeucalenol, cyclolaudenol, desmosterol, episterol, ergosterol, isofucosterol, schottenol, sitostanol, spinasterol and stigmasterol. The m/z and retention time for each sterol is shown in Table S2. The sterol composition of all samples was signal-corrected by dividing the signal area for each metabolite by the total area of all Level 3 annotated signals for that sample, rendered per mille (‰). The internal standard (*d*_*7*_-cholesterol; 20 μL of 0·1 mg/mL, 5·084 μMoles/sample; doped into all pollen samples before saponification) and mass of pollen was used to calculate the total sterol the pollen per mg. However, data from this calculation were not used.

**Sterol grouping.** The combination of the array of sterols measured and the different sterol biosynthesis pathways described (Fig. 1) led us to suggest that sterols found in plants can be grouped by the number of carbons in the sterol. This grouping is useful for interpreting sterolome readouts as differences in sterol composition between species indicate differences in which enzyme-catalysed reactions in the sterol pathway are faster/slower. This shows how the biochemistry of the system differs between taxonomical groups and was used in the present study to simplifying the dataset in order to plot a readily readable heatmap (Fig. 2A).

### 5.3 Evolution of sterol metabolism

Plant tissue comparisons. We used the relative abundance of sterols to determine semi-quantitative (dis)similarities by calculating the Bray-Curtis similarity index. Then a permutational multivariate analysis of variance (PERMANOVA) with 10 000 permutations based on the (dis)similarities was performed to test for statistical differences in sterol composition between leaves and pollen while considering plant tissue as a fixed effect and species as a random effect. A similarity percentage (SIMPER) analysis was also performed to identify the main sterol driving potential differences. We performed a principal component analysis (PCA) to show sterolome differences between samples graphically and identify the variables that drive the difference.

Pollination type comparisons. To evaluate differences in sterol composition in pollen between wind- and animal-pollinated plants and the patterns therein, we used only pollen samples. As above, (dis)similarities were calculated and employed for PERMANOVA, where pollination strategy (wind or animal pollinated) and taxonomic order were used to account for potential similarities in sterol composition among closely related taxa and were considered as fixed effects. A PCA and a SIMPER were also performed for graphical display and identifying the main differentiating sterols, respectively.

Pollinator type and reward comparisons. To evaluate whether the sterol composition of pollen differed between plants with different types of pollination (bee, butterfly, moth, generalist) and offering different types of reward (*i.e.* pollen, nectar and oil), we tested pollen samples of animal pollinated plants only. PERMANOVA and SIMPER were carried out. The taxonomic order and type of pollinator and reward were considered fixed effects in the PERMANOVA. *Post hoc* tests for type of pollinator and reward were implemented after the PERMANOVA. These analyses were performed using the vegan (Oksanen et al., 2019) and pairwiseAdonis (Martinez Arbizu, 2017) packages of R v.3.6.32 017.

Ancestral state reconstruction. We reconstructed the phylogenetic relationships among 31 of the sampled species (Table 1), for which we have both pollen and leaf data using the package U. phyloMaker (Jin and Qian, 2022) of R and based on an updated mega-tree of vascular plants ‘GBOTB.extended.tre’ (Smith and Brown, 2018). In order to understand the evolutionary trajectory of the main sterols driving differences among organs and pollination mechanisms, we reconstructed the ancestral state for the continuous characters “proportions of campesterol, cycloartenol, desmosterol, isofucosterol, 24MC, and *beta*-Sitosterol” using the reconstructed phylogenetic tree. Then, a Maximum Likelihood estimation of continuous characters was carried out with the reconstructed tree using the phytools package of R (Revell, 2012).

## Author contributions

SF and HK conceived the project and designed experiments. AY, SF and HK collected samples. SF and HK extracted samples. SF developed LCMS methods and data collection. SF ran samples and processed data. CM analysed data and conducted statistical tests with SF. HK, SF and GCS supervised AY. SF, HK and PCS supervised the project and interpreted data with CM. SF wrote and revised the manuscript with comments from all authors. All authors commented on the manuscript and approved the final version. PCS and HK devised the overarching research questions and wrote the original grant proposals that funded the work.

## Declaration of competing interest

The authors declare that they have no known competing financial interests or personal relationships that could have appeared to influence the work reported in this paper.

## Data Availability

Data will be made available on request.
