# Supplementary material for: Sterol composition in plants is specific to pollen, leaf, pollination and pollinator
Source: Phytochemistry. 2023 Oct;214:None. doi: 10.1016/j.phytochem.2023.113800 (PMC10493607; doi:10.1016/j.phytochem.2023.113800)
Supplement: Multimedia component 1 [file mmc1.docx]

**Supplementary Figures**

A

B

**Fig. S1. The relative abundance of C_27_, C_28_, C_29_, C_30_ and C_31_ sterols in pollen samples from wind- and animal-pollinated species.** Panel **A**, sterol profiles from animal-pollinated plants; **B**, sterol profiles from wind-pollinated plants.

|  | Leaves | | Pollen | | |
| --- | --- | --- | --- | --- | --- |
| No.  carbons | Freq | Freq >60% of the total | Freq | Freq >60% of the total |  |
| 27 | 0 | 0 | 2 | 2 |  |
| 28 | 0 | 0 | 4 | 2 |  |
| 29 | 27 | 17 | 23 | 19 |  |
| 30 | 4 | 4 | 2 | 1 |  |
| 31 | 0 | 0 | 0 | 0 |  |
| Total | 31 | 21 | 31 | 24 |  |

**Table S1. The frequency of plant species by dominant sterol type (the number of carbon atoms in the sterols found) and where that sterol type makes up over 60% of the total sterol signal (relative abundance).**

| ID | Configuration | Molecular formula | Exact mass (*m/z*) | M-[HO-] | M+H | Delta | Rt  (min) |
| --- | --- | --- | --- | --- | --- | --- | --- |
| 24-methylenecholesterol | ST(28:2) | C_28_H_46_O | 398.3549 | 381.3516 | 399.3622 | 5 | 4.85 |
| 24-methylenecycloartanol | ST(30:1)pr | C_30_H_50_O | 426.3862 | 409.3829 | 427.3935 | 0 | 7.49 |
| Anthelsterol* | ST(29:3) | C_29_H_46_O | 410.3594 | 393.3561 | 411.3667 |  | 4.41 |
| Avenasterol | ST(29:2) | C_29_H_48_O | 412.3705 | 395.3672 | 413.3778 | 7 | 6.20 |
| Brassicasterol | ST(28:2) | C_28_H_46_O | 398.3549 | 381.3516 | 399.3622 | 5 | 5.60 |
| Campesterol | ST(28:1) | C_28_H_48_O | 400.3705 | 383.3672 | 401.3778 | 5 | 6.87 |
| Cholesterol | ST(27:1) | C_27_H_46_O | 386.3549 | 369.3516 | 387.3622 | 5 | 5.59 |
| Cycloartenol | ST(30:1)pr | C_30_H_50_O | 426.3862 | 409.3829 | 427.3935 | 0 | 7.20 |
| Cycloeucalenol | ST(30:1)pr | C_30_H_50_O | 426.3862 | 409.3829 | 427.3935 | 0 | 6.62 |
| Cyclolaudenol | ST(31:1)pr | C_31_H_52_O | 440.4018 | 423.3985 | 441.4091 | 0 | 8.50 |
| *d_7_*-cholesterol | ST(27:1) | C_27_H_39_D_7_O | 393.399 | 376.3957 | 394.4063 | 5 | 5.59 |
| Desmosterol | ST(27:2) | C_27_H_44_O | 384.3392 | 367.3359 | 385.3465 | 5 | 4.20 |
| Episterol | ST(28:2) | C_28_H_46_O | 398.3549 | 381.3516 | 399.3622 | 7 | 4.61 |
| Ergosterol | ST(28:3) | C_28_H_44_O | 396.3392 | 379.3359 | 397.3465 | 5,7 | 4.55 |
| Isofucosterol | ST(29:2) | C_29_H_48_O | 412.3705 | 395.3672 | 413.3778 | 5 | 5.99 |
| Schottenol | ST(29:1) | C_29_H_50_O | 414.3862 | 397.3829 | 415.3935 | 7 | 7.80 |
| Sitostanol | ST(29:0) | C_29_H_52_O | 416.4018 | 399.3985 | 417.4091 | 0 | 9.20 |
| *beta-*Sitosterol | ST(29:1) | C_29_H_50_O | 414.3862 | 397.3829 | 415.3935 | 5 | 8.17 |
| Spinasterol | ST(29:2) | C_29_H_48_O | 412.3705 | 395.3672 | 413.3778 | 7 | 6.73 |
| Stigmasterol | ST(29:2) | C_29_H_48_O | 412.3705 | 395.3672 | 413.3778 | 5 | 7.20 |

**Table S2. A list of the sterols identified in this study, with retention times and *m/z* values. *name given for convenience in this study as the sterol was isolated from *Anthelmintica*.**
